# Study of Asymmetric Test Configurations by Means of Standard and Revised Virtual Crack-Closure Techniques

**DOI:** 10.3390/ma19112421

**Published:** 2026-06-05

**Authors:** Jorge Bonhomme, Victoria Mollón

**Affiliations:** 1 Department of Construction and Manufacturing Engineering, University of Oviedo, 33203 Gijón, Spain; 2 Department of Materials Science and Metallurgical Engineering, University of Oviedo, 33203 Gijón, Spain; mollonvictoria@uniovi.es

**Keywords:** VCCT, mixed-mode delamination, AENF, ADCB

## Abstract

**Highlights:**

- The standard VCCT results fall between the revised VCCT results in ADCB and AENF.
- Contact between the specimen arms can lead to unrealistic negative VCCT values.
- In the revised VCCTs, ERR transfer occurs between modes I and II.

**Abstract:**

The objective of this article is to compare the standard two-step virtual crack-closure technique (VCCT) and the revised I–II and II–I VCCT developed by Valvo by studying two asymmetric test configurations commonly used to produce mixed-mode delamination in composite laminates—the asymmetric double cantilever beam (ADCB) and asymmetric end-notched flexure (AENF) configurations—via finite element modelling (FEM). Scientific literature has revealed that highly asymmetric specimens may exhibit negative components of the energy release rate (ERR) under certain specific loading conditions when using the standard VCCT. The revised VCCTs establish an alternative ERR partition with energetically orthogonal components to solve this inconsistency. This study aims to better understand the mechanisms involved in the revised VCCTs. This study demonstrates that, when using the revised methods, there is a transfer of energy between modes I and II, unlike when using the standard VCCT. The values of the mode I and mode II components of the ERR produced by the standard VCCT fall between the values produced by the revised I–II and II–I VCCTs for the test configurations. Nevertheless, as expected, the total ERR calculated using the three procedures is the same. Finally, some considerations are drawn for the scenario when contact occurs between the specimen arms in the AENF configuration, as it can also lead to unrealistic negative mode I ERR values in the FEM analysis.

## 1. Introduction

### 1.1. Standard VCCT

Delamination in composite laminates is usually characterised in terms of the energy release rate (ERR). The ERR (*G*) is defined as the total energy released per unit area when the crack extends by a length *da*. In mathematical form:

(1) $$G=-\frac{1}{B}\frac{dU}{da}$$

where *U* is the internal energy, *a* is the crack length, and *B* is the specimen width.

Crack initiation and growth in laminated composites can occur under pure loading modes I, II, and III. A combination of these modes is usually encountered in practice. *G_I_*, *G_II_*, and *G_III_* represent the ERR components under loading modes I, II, and III, respectively. In two-dimensional (2D) problems under mixed-mode I/II, the total ERR *G* can be partitioned as *G* = *G_I_* + *G_II_*.

The virtual crack-closure technique (VCCT) [1,2] is currently one of the numerical methods most widely used to study delamination fractures in composite laminates. This method is based on the premise that the energy required to extend the crack by a given distance Δ*a* is the same as the energy required to close the crack by the same distance. This method was first implemented numerically by Rybicki and Kanninen [3] and is based on earlier work by Irwin [4].

**Figure 1:**
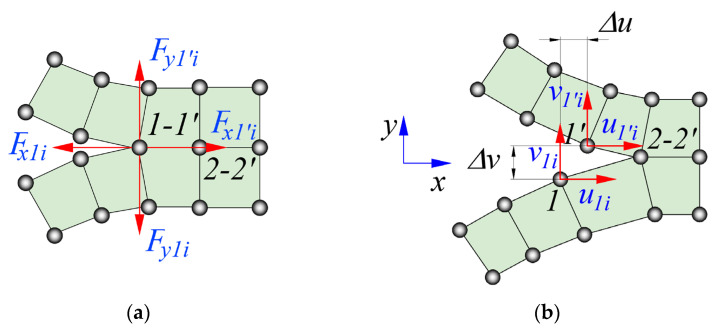
Two-step standard VCCT. The subscript *i* considers the extension to a 3D model with *i* nodes at the crack front. (**a**) Nodes 1–1′are constrained in the vertical and horizontal directions. (**b**) Horizontal and vertical release of nodes 1–1′.

Figure 1 shows the two-step VCCT, also known as the two-step extension method. In this method, the crack path is modelled by coincident nodes of the upper and lower sub-laminates, in which the degrees of freedom of each pair of nodes are coupled. In the first step, the specimen is loaded and the forces at the crack tip are calculated for nodes 1–1′ (see Figure 1a).

In the second step (Figure 1b), the constraints holding these two nodes together are released and the displacements are calculated for the same node pair. *F_j_*_1*i*_ and *F_j_*_1′*i*_ represent the forces acting on the crack tip (nodes 1–1′). The subscript *i* considers the extension to a three-dimensional (3D) model with *i* nodes at the crack front. These forces are due to the action of an external force applied to the specimen. As nodes 1–1′ are coupled in step (a), *F_x_*_1*i*_ = −*F_x_*_1′*i*_ and *F_y_*_1*i*_ = −*F_y_*_1′*i*_. On the other hand, *u*_1*i*_, *u*_1′*i*_, *v*_1*i*_, and *v*_1′*i*_ are the horizontal and vertical displacements of nodes 1 and 1′ that occur when the crack tip nodes are released.

*G_I_* and *G_II_* are then calculated as follows (considering the force acting on the upper node as the reference):

(2) $$G_{I}=\frac{1}{2B∆a}\sum_{i=1}^{n}F_{y1^{\prime }i}(v_{1^{\prime }i}-v_{1i})=\frac{1}{2B∆a}\sum_{i=1}^{n}F_{y1^{\prime }i}∆v_{i}$$

(3) $$G_{II}=\frac{1}{2B∆a}\sum_{i=1}^{n}F_{x1^{\prime }i}\left(u_{1^{\prime }i}-u_{1i}\right)=\frac{1}{2B∆a}\sum_{i=1}^{n}F_{x1^{\prime }i}∆u_{i}$$

For a 2D model under plane strain conditions, and ignoring the subscripts 1–1′, which refer to the pair of nodes at which the forces and displacements are calculated, these equations can be re-written in a simplified form.

(4) $$G_{I}=\frac{1}{2B∆a}F_{y}∆v$$

(5) $$G_{II}=\frac{1}{2B∆a}F_{x}∆u$$

**Figure 2:**
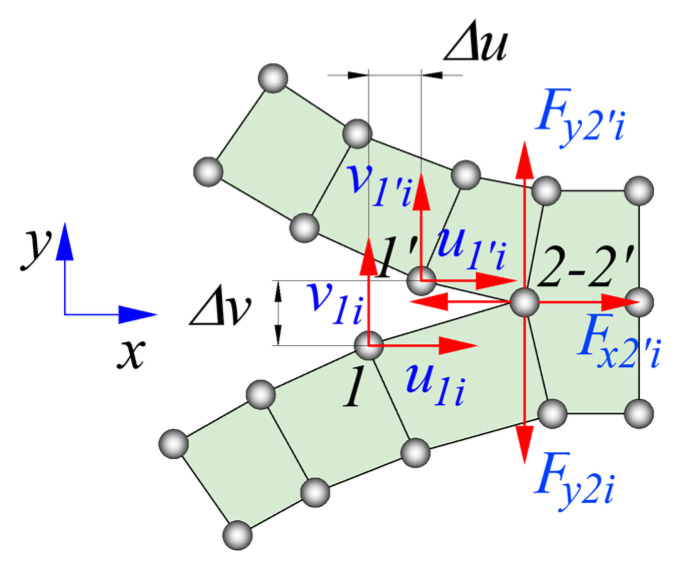
One-step standard VCCT.

However, in commercial finite element modelling (FEM) packages, VCCT is usually implemented as a simplified one-step method [1,5], which is more efficient from a computational point of view. In this case, the forces and displacements are calculated in the same step, in two different pairs of nodes, 1–1′ and 2–2′ (see Figure 2). This simplification is valid as long as the crack increment Δ*a* is sufficiently small.

In this case, the ERR calculation is performed as follows (considering the force acting on the upper node as the reference):

(6) $$G_{I}=\frac{1}{2B∆a}\sum_{i=1}^{n}F_{y2^{\prime }i}(v_{1^{\prime }i}-v_{1i})$$

(7) $$G_{II}=\frac{1}{2B∆a}\sum_{i=1}^{n}F_{x2^{\prime }i}\left(u_{1^{\prime }i}-u_{1i}\right)$$

The VCCT-based crack growth simulations involve the assumptions that the analysis is quasi-static, does not account for transient effects and that the material is linear elastic [5].

### 1.2. Revised VCCTs

Valvo [6,7] and Wang et al. [8] observed that, in asymmetric laminates under certain loading conditions, VCCT could erroneously predict negative values for *G_I_* and *G_II_*. The authors of this article have also occasionally detected slightly negative *G_I_* values in some configurations with a high degree of asymmetry [9]. Asymmetry can occur for several reasons: when the crack plane is not located in the plane of symmetry of the specimen, when the elastic properties of the sub-laminates are different, or under certain loading conditions.

In his articles [6,7], Valvo drew attention to the fact that the VCCT equations shown above did not define *G_I_* and *G_II_* as positive definite quantities. They could be negative when the nodal forces and their corresponding displacements were in opposite directions.

In asymmetric systems, the application of horizontal forces can cause vertical displacements of nodes, while the application of vertical forces can cause horizontal movements of those nodes. This means that interactions may occur between modes I and II. To visualise this coupling, Valvo introduced flexibility coefficients *f_ij_* [6,7], where *i*, *j* ∈ {*x*, *y*}, to calculate the relative node displacements produced by crack extension.

(8) $$∆u=f_{xx}F_{x}+f_{xy}F_{y}$$

(9) $$∆v=f_{yx}F_{x}+f_{yy}F_{y}$$

Δ*u* and Δ*v* are the horizontal and vertical relative displacements. *F_x_* and *F_y_* are the horizontal and vertical forces acting on the nodes. Based on energetic considerations, Valvo [6,7] established that *f_xx_* and *f_yy_* were always positive. However, *f_xy_* = *f_yx_* could be positive or negative, being zero in symmetrical specimens.

To solve this inconsistency, Valvo proposed a revised VCCT [6] with the goal of obtaining a physically consistent *G_I_*/*G_II_* partition with always non-negative partition values. In his article, Valvo [6] defined the procedure in the crack-closure direction. This method, described in the crack-opening direction, is as follows (there is no physical difference when defining this process in the opening or closing direction, producing the same results as it is a reversible process).

**Figure 3:**
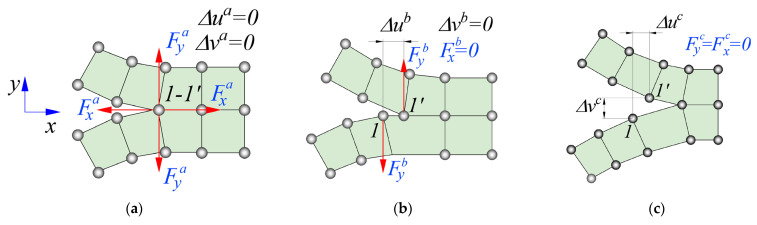
Revised II–I VCCT. (**a**) Nodes 1–1′are constrained in the vertical and horizontal directions. (**b**) Horizontal release of nodes 1–1′. Pure mode II is produced. (**c**) Vertical release of nodes 1–1′. Pure mode I is produced.

In the first step, a prescribed displacement *δ* is applied to the specimen. Owing to the node-coupling constraints (Δ*u^a^* = Δ*v^a^* = 0), horizontal and vertical forces (*F_x_^a^*, *F_y_^a^*) appear in the node pair 1–1′ (see Figure 3a). In the second step (see Figure 3b), the horizontal coupling in nodes 1–1′ is released (*F_x_^b^* = 0) while maintaining the vertical restriction (Δ*v^b^* = 0). The crack tip nodes are then allowed to move horizontally by a relative amount Δ*u^b^*, so that pure mode II is produced. *G_II_* is then calculated from the horizontal force *F_x_^a^* and relative horizontal displacement Δ*u^b^*. In this step, *G_I_* = 0 because of the zero vertical relative displacement (Δ*v^b^* = 0).

Note that in the second step (Figure 3b), a reaction force *F_y_^b^* appears at nodes 1–1′ (*F_y_^b^* ≠ *F_y_^a^*) because the vertical movement of the nodes is restricted (Δ*v^b^* = 0). In the third step (Figure 3c), the vertical restriction is released; therefore, the nodes now move vertically (Δ*v^c^*) and horizontally (Δ*u^c^* − Δ*u^b^*). From steps (b) to (c), only the vertical force *F_y_^b^* is effective; therefore, pure mode I exists. Although a relative horizontal movement Δ*u^c^* − Δ*u^b^* of the crack tip nodes is produced by the vertical release of the nodes, as *F_x_^b^* = 0, *G_II_* = 0 in this step.

Under these conditions, the energy partition is as follows.

(10) $$G_{II}=\frac{1}{2B∆a}F_{x}^{a}∆u^{b}$$

(11) $$G_{I}=\frac{1}{2B∆a}F_{y}^{b}∆v^{c}$$

As this procedure, developed by Valvo [6], produces pure mode II first, and then pure mode I in the crack-opening direction, it will be referred to in this article as ‘Revised II–I VCCT’.

This partitioning mode ensures that *G_I_* and *G_II_* are always positive, as demonstrated by Valvo in terms of the flexibility coefficients [6].

As the initial and final states of the model are the same in the standard and revised partitions, the total energy released during crack growth will be the same in both procedures. Moreover, comparing Figure 1 and Figure 3, we reveal that *F_x_^a^* = *F_x_*, *F_y_^a^* = *F_y_*, Δ*u^c^* = Δ*u*, and Δ*v^c^* = Δ*v*.

This procedure can then be inferred to have been based on the following premises.

- Pure mode II is obtained when the crack tip opening displacement Δ*v* is zero.
- Pure mode I is obtained when the tangential crack tip force *F_x_* is zero.

In a later article [7], Valvo questioned the above premises and proposed an alternative:

- Pure mode I is obtained when the tangential crack tip displacement Δ*u* is zero.
- Pure mode II is obtained when the crack tip opening force *F_y_* is zero.

**Figure 4:**
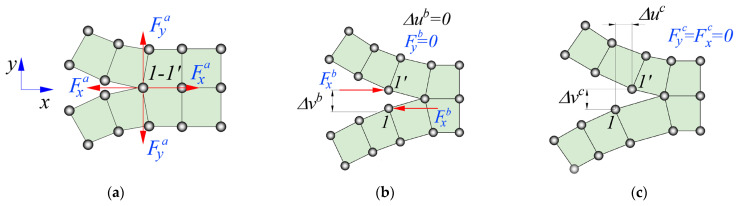
Revised I–II VCCT. (**a**) Nodes 1–1′are constrained in the vertical and horizontal directions. (**b**) Vertical release of nodes 1–1′. Pure mode I is produced. (**c**) Horizontal release of nodes 1–1′. Pure mode II is produced.

These alternative premises are presented in Figure 4 and described below.

In the first step, a prescribed displacement *δ* is applied to the specimen (Figure 4a). Therefore, horizontal and vertical forces (*F_x_^a^*, *F_y_^a^*) appear in the node pair 1–1′ because of the node-coupling constraints (Δ*u^a^* = Δ*v^a^* = 0). In the second step (Figure 4b), the vertical constraint in nodes 1–1′ is released, maintaining the horizontal restriction (Δ*u^b^* = 0). The crack tip nodes are then allowed to move vertically by a relative amount equal to Δ*v^b^*, producing pure mode I. *G_I_* is then calculated from the released force *F_y_^a^* and relative vertical displacement Δ*v^b^*. In this step, *G_II_* = 0 because the horizontal relative displacement is zero (Δ*u^b^* = 0).

Note that in the second step (Figure 4b), as the horizontal movement of the nodes is restricted (Δ*u^b^* = 0), a reaction force *F_x_^b^* appears in nodes 1–1′ (*F_x_^b^* ≠ *F_x_^a^*). In the third step (Figure 4c), the horizontal constraint is released. Therefore, the nodes are now allowed to move horizontally (Δ*u^c^*) and vertically (Δ*v^c^* − Δ*v^b^*). From (b) to (c) as *F_y_^b^* = 0, only the horizontal force *F_x_^b^* is effective, and pure mode II is produced (*G_II_*).

Under these conditions, the energy partition is as follows.

(12) $$G_{I}^{b}=\frac{1}{2B∆a}F_{y}^{a}∆v^{b}$$

(13) $$G_{II}^{c}=\frac{1}{2B∆a}F_{x}^{b}∆u^{c}$$

As this procedure, developed by Valvo [7], produces pure mode I first, and then pure mode II in the crack-opening direction, it will be referred to in this article as ‘Revised I–II VCCT’.

These two procedures (revised I–II and II–I VCCT) avoid the interactions between the vertical forces and horizontal displacements, and conversely, the interactions between the horizontal forces and vertical displacements.

It should be noted that, when analysing mixed-mode delamination in asymmetric laminates, in most cases the standard VCCT method provides physically consistent *G_I_* and *G_II_* positive values. Only on certain occasions, under specific loading conditions and with moderate to high asymmetry ratios, may slightly negative values of *G_I_* or *G_II_* appear. In his study [6], Valvo analysed an ADCB specimen subjected to bending moments *M*_1_ and *M*_2_ at the free ends of the sub-laminates. Valvo found slightly negative values of *G_I_* and/or *G_II_* for asymmetry ratios of *h*_1_/*h*_2_ = 1/4 and *h*_1_/*h*_2_ = 1/19, for certain applied bending moments *M*_1_/*M*_2_ ratios. Furthermore, as the degree of asymmetry increased, the difference between the standard VCCT and the revised VCCTs increased.

Wang et al. [8,10,11,12,13] also studied the partition theories in depth. From mechanical considerations, they defined two orthogonal pairs of locally pure modes I and II. The first pair corresponded to zero relative shear displacement at the crack tip Δ*u* = 0 (to produce pure mode I) and zero crack tip opening force *F_y_* = 0 (to produce pure mode II). They named it *DF*-(*θ*, *β*). In this nomenclature, *D* indicates that the pure mode is due to zero displacement, and *F* indicates that the pure mode is due to zero crack tip force [10]. The second pair, named as *FD*-(*θ*′, *β*′), corresponded to zero crack tip shearing force *F_x_* = 0 (to produce pure mode I) and zero crack tip opening displacement Δ*v* = 0 (to produce pure mode II) [10]. These conditions were equivalent to those established by Valvo, which are explained above.

Xu et al. [14] have recently studied the revised VCCT methods developed by Valvo and have proposed a new method to avoid having to perform the calculation in two steps. This method, named the physical VCCT, decomposes the mixed mode into positive components, performing the calculations in a single step using a rotated coordinate system. Xu et al. compared this physical VCCT with the classical VCCT and Valvo’s revised VCCTs. They concluded that the physical VCCT provides unique and positive partition values based on a solid physical foundation. Other authors have taken a different approach to improve the performance of the classical VCCT by combining VCCT with other methods, such as the enriched finite element combined with the VCCT method (EFEM-VCCT) proposed by Zhou et al. [15]. According to these authors, the EFEM-VCCT reduces the mesh quality requirements and improve the solution accuracy compared to the classical VCCT.

In this article, the standard two-step, revised I–II, and revised II–I VCCTs are programmed using the ANSYS parametric design language (APDL) in order to study two asymmetric testing configurations commonly used to produce I/II mixed-mode delamination—the asymmetric double cantilever beam (ADCB) and asymmetric end-notched flexure (AENF) configurations—via FEM. The aim of this study is to compare the three VCCTs and provide insight on the mechanisms involved in the revised procedures. Some considerations are also drawn regarding the contact that occurs between the specimen arms in the AENF configuration.

For simplicity, 2D models were used in this work; nevertheless, the conclusions can be directly extended to 3D models.

## 2. Materials and Methods

**Figure 5:**
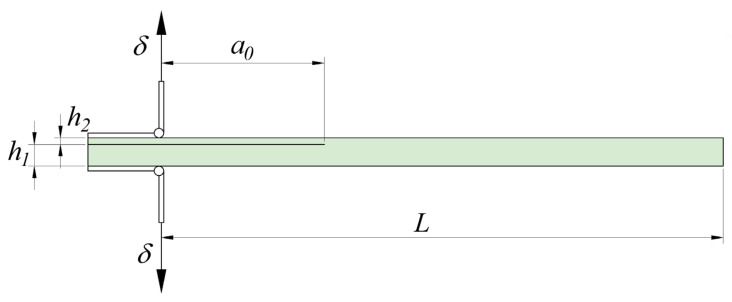
ADCB test configuration, where *δ*: applied displacement; *h*_1_, *h*_2_: sub-laminate thickness; *a*_0_: initial crack length; *L*: total length.

The partition theories mentioned above were studied using two test configurations. One of the studied configurations was the ADCB (see Figure 5) [16,17,18]. In this test configuration, both sub-laminates had different stiffnesses (different thickness or different elastic properties), resulting in a mixed mode at the crack tip. In this test configuration, mixed-mode I/II was generated with a predominance of mode I.

This test configuration is similar to the standardised double cantilever beam (DCB) [19,20], with the difference that the crack plane lies outside the midplane. The displacement *δ* is applied using hinges. The dimensions of the specimen and the applied displacement *δ* are given below.

**Figure 6:**
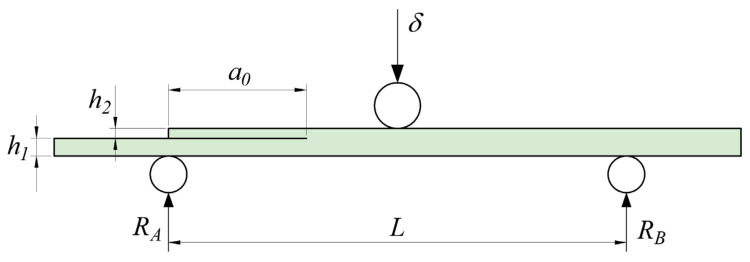
AENF test configuration, where *δ*: applied displacement; *h*_1_, *h*_2_: sub-laminate thickness; *a*_0_: initial crack length; *L*: span length.

The second test configuration studied in this work was the AENF [21] (see Figure 6). As in the ADCB test specimen, both sub-laminates had different stiffnesses. In this case, a mixed mode was present at the crack tip when the stiffer sub-laminate was placed on the top, while pure mode II occurred when the stiffer sub-laminate was placed at the bottom [9]. In this test configuration, mixed-mode I/II was generated, with a predominance of mode II.

This test configuration is similar to the standardised ENF [22], with the difference that the crack plane lies outside the midplane. The dimensions of the loading and support cylinders were 5 mm and 4 mm, respectively. The dimensions of the specimen and the applied displacement *δ* are given below.

As can be seen in Figure 6, the upper leg was trimmed to avoid interference between the edges when the specimen flexed under the applied load, as indicated by Sundararaman et al. [21].

In order to perform this study, 2D FEM models with plane strain behaviours were prepared using ANSYS^®^ 2024 Academic Research software. The element used to mesh the model was the 2D four-node structural solid PLANE182 with full integration and pure displacement formulation options. Several tests were conducted, progressively decreasing the element size to optimise the mesh. Finally, a regular mesh size of 0.05 mm was selected. A finer mesh would produce a variation in the ERR values of less than 0.1%.

The final ADCB model consisted of 360,000 elements and 366,122 nodes, and the final AENF model consisted of 378,680 elements and 378,699 nodes. A specimen width of *B* = 1 mm was assumed throughout the study.

In these models, the crack path was modelled using pairs of coincident nodes that were initially coupled to each other (joined). The forces at the crack tip were calculated in a first step when the applied displacement reached a prescribed value. The imposed displacement in the specimen was then held, and the coupled degrees of freedom (DOFs) of the nodes at the crack tip were released in the second step. The ERR was then calculated by means of the released forces and the resulting displacements. In the standard VCCT, the vertical and horizontal constraints were released simultaneously, while in the revised VCCTs the process was performed in two steps, releasing only one of the constraints in each step.

The material used in the models was a unidirectional carbon fibre-reinforced epoxy laminate (CFRP). The elastic properties assumed for this material are shown in Table 1 [9].

**Figure 7:**
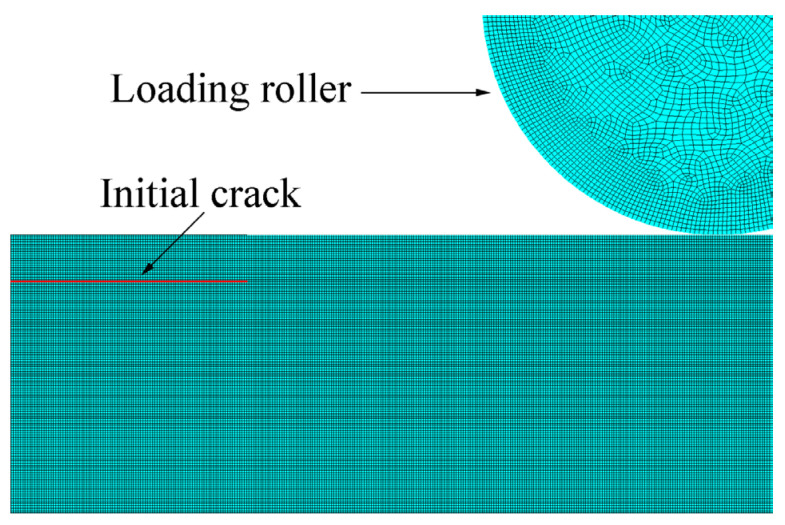
AENF FEM model.

The models were solved using the three mentioned procedures (standard two-step, revised I–II, and revised II–I VCCTs) to calculate *G_I_* and *G_II_*. For this purpose, specific Ansys APDL scripts were prepared. The scripts were programmed in the crack-opening direction, as described in the previous section. Figure 7 shows a detail of the AENF model.

This study was limited to linear elastic materials. Linear elastic fracture mechanics (LEFM) was assumed throughout this article.

## 3. Results and Discussion

### 3.1. ADCB Specimen

The first FEM model studied in this work was the ADCB specimen. The dimensions of the model were as follows (see Figure 5):

- *L* = 150 mm;
- *a*_0_ = 50 mm;
- *h*_1_ = 4 mm;
- *h*_2_ = 2 mm.

A displacement of *δ* = 3 mm was applied to all the models. The geometry of the specimen and applied displacement were selected so as to avoid excessive rotation of the specimen and its influence on the results [23].

**Figure 8:**
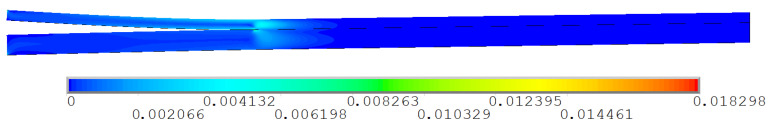
ADCB specimen; von Mises elastic strain at an applied displacement of *δ* = 3 mm.

This model was analysed using the standard two-step, revised I–II, and revised II–I VCCTs. Figure 8 shows the deformed shape of the specimen together with the von Mises elastic strain map. The obtained results are discussed below.

#### 3.1.1. Standard Two-Step VCCT

The model was first solved using the standard two-step VCCT. Table 2 shows the obtained results. These results were considered as a reference for later comparisons with the revised I–II and II–I procedures. The last column of this table shows the total ERR *G* calculated from the variation in the internal energy of the system (Equation (1)) for comparison with the VCCT results (the error is shown in brackets). This table shows the forces acting on the upper node. As the nodes at the crack tip were coupled together before crack extension, *F_bottom_* = −*F_Top_*.

As can be observed in Table 2, the total ERR calculated through VCCT and through the variation in internal energy were in good agreement.

#### 3.1.2. Revised I–II VCCT

In a second run, the FEM model was solved following the revised I–II procedure (Figure 4). The results are presented in Table 3. This table shows the forces acting on the upper node. Table 4 shows a comparison of the standard two-step and revised I–II VCCT.

As can be seen in Table 3, when going from step (a) to (b), only pure mode I is produced, while when going from step (b) to (c), only pure mode II takes place. Although the nodes move vertically by Δ*v^c^* in the last step, mode I ERR is not produced as *F_y_^b^* = 0.

Comparing the I–II revised procedure (Table 3) with the standard VCCT (Table 2), we note that *F_x_^a^* = *F_x_*, *F_y_^a^* = *F_y_*, Δ*u^c^* = Δ*u*, and Δ*v^c^* = Δ*v*. As the initial and final steps in both procedures are the same, the total energy *G* must be the same in both. Only the partition *G_I_*/*G_II_* differs from one procedure to another (see Table 4).

When comparing the mode I ERR provided by the revised I–II VCCT with that of the standard VCCT, a decrease of −4.6 N/m is observed in the *G_I_* value (Table 4). This loss in *G_I_* is equivalent to:

(14) $$G_{I\;}=\frac{1}{2B∆a}F_{y}^{a}\left(∆v^{c}-∆v^{b}\right)=-4.6\;N/m$$

The decrease in the *G_I_* value is recovered by the increase in the horizontal force when going from (a) to (b) (*F_x_^a^* = −9.40 N increases to *F_x_^b^* = −10.22 N). Thus, the mode II work produced in the last step of revised I–II VCCT exceeds that produced in the standard model by a quantity equal to:

(15) $$G_{II\;}=\frac{1}{2B∆a}\left({F_{x}^{b}-F}_{x}^{a}\right)∆u^{c}=4.7\;N/m$$

This increase in *G_II_* is practically equal to the magnitude of the loss in the *G_I_* value (−4.6 N/m). This is equivalent to an ERR transfer from mode I to mode II, keeping the total ERR *G* unchanged.

As pure modes occur in each step of the revised I–II VCCT, the use of the variations in the internal energy is a valid alternative method to the VCCT for calculating *G_I_* and *G_II_* in each step, since all energy variations can be attributed exclusively to mode I or mode II in each step.

#### 3.1.3. Revised II–I VCCT

The FEM model was next solved following the revised II–I VCCT (see Figure 3). The results are presented in Table 5.

Table 6 shows a comparison between the standard two-step and revised II–I VCCT.

As we can see in Table 5, when going from step (a) to (b) (Figure 3), only pure mode II is produced, while when going from step (b) to (c), only pure mode I is produced.

Another transfer of ERR between the fracture modes occurs. In this case, the ERR transfer takes place from mode II to mode I. As can be seen in Table 6, the decrease in the mode II component of the ERR practically coincides with the increase in the mode I component (the slight difference can be attributed to minor numerical errors).

When going from (a) to (b), there is a decrease in mode II ERR compared to that observed in the standard procedure, which is equivalent to:

(16) $$G_{II}=\frac{1}{2B∆a}F_{x}^{a}\left(∆u^{c}-∆u^{b}\right)=-4.0\;N/m$$

Nevertheless, when going from (a) to (b), the vertical force acting on the crack tip nodes increases (*F_y_^a^* = 11.89 N increases to *F_y_^b^* = 12.03 N), storing energy in this process. In the next step, from (b) to (c), this vertical force is released, producing mode I ERR. In this process, an ‘additional’ mode I ERR is produced, which is equivalent to:

(17) $$G_{I\;}=\frac{1}{2B∆a}\left({F_{y}^{b}-F}_{y}^{a}\right)∆v^{c}=4.1\;N/m$$

Table 7 shows a summary of the standard two-step, revised I–II, and revised II–I VCCTs.

Table 7 indicates that the *G_I_* value provided by the standard model is nearly the midpoint of the *G_I_* results of the revised I–II and II–I models, with the higher value being produced by the II–I model. The *G_II_* value furnished by the standard model is also centred between the results of the revised models; however, in this case, it is the revised model I–II that produces the higher value of *G_II_*. The total ERR *G* is the same in all three models.

As can be seen from the above calculations, in the revised I–II model, a certain amount of mode I ERR is transferred to mode II ERR. In the II–I model, the opposite occurs: a portion of the energy from mode II is transferred to mode I.

#### 3.1.4. Influence of the Degree of Asymmetry

**Figure 9:**
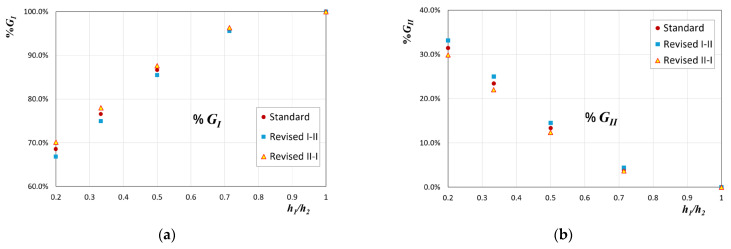
ADCB test. Influence of the degree of asymmetry: (**a**) *G_I_* and (**b**) *G_II_*.

Some ADCB models were prepared with different degrees of asymmetry. In all models, a displacement of 3 mm was applied to the specimen lips. The partition values were calculated using the three VCCTs. Figure 9 shows the obtained results.

As can be seen in Figure 9, for symmetric specimens (*h*_1_/*h*_2_ = 1), the results of the three calculation procedures are coincident. For asymmetric specimens, there is a slight difference between the partition calculations, with the results of the standard procedure being centred between those of the revised procedures.

### 3.2. AENF Specimen with Lower Arm Stiffer than Upper Arm

The next model studied in this work is the AENF test specimen.

As shown in [9], the positioning of the AENF test specimen determines whether the mixed-mode I/II or pure mode II is obtained at the crack tip. When the stiffer laminate is placed at the bottom, pure mode II occurs, while when it is placed at the top, mixed-mode I/II is produced.

The dimensions of the model used in this study are as follows (see Figure 6):

- *L* = 100 mm;
- *a*_0_ = 40 mm;
- *h*_1_ = 5 mm;
- *h*_2_ = 1 mm.

In this case, the lower arm is stiffer than the upper arm; therefore, pure mode II is produced at the crack tip.

In this test configuration, contact occurs between the laminate arms and between the laminate and the loading and support cylinders. Therefore, such contact interactions must be modelled in order to avoid mesh interpenetration. The contact surfaces were modelled using TARGE169 and CONTA172 elements. The contact algorithm used was the augmented Lagrange method without friction.

**Figure 10:**
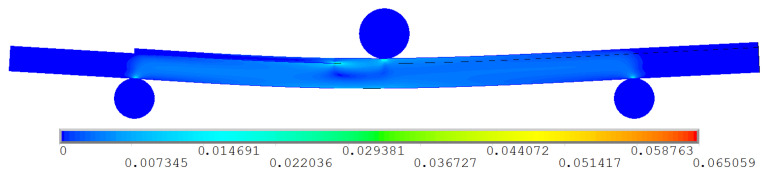
AENF specimen; von Mises elastic strain at an applied displacement of *δ* = 2 mm.

This model was analysed using the standard two-step, revised I–II, and revised II–I VCCTs. Specific APDL scripts were developed for this purpose. The mechanical properties of the materials were taken from Table 1. A displacement of *δ* = 2 mm was applied to the specimen midpoint. Figure 10 shows the deformed shape of the specimen together with the von Mises elastic strain map. The obtained results are discussed below.

#### 3.2.1. Standard Two-Step VCCT

Table 8 shows the obtained standard two-step VCCT results. The last column of this table shows the total ERR *G* calculated from the variation in the internal energy of the system (Equation (1)) for comparison with the VCCT results.

As can be observed in Table 8, the standard two-step VCCT provides an inconsistent small negative value for *G_I_*. When solving this model with the one-step VCCT implemented in the Ansys package using the Ansys function CINT, TYPE, VCCT, similar results were obtained. As mentioned above, a negative *G_I_* value has no physical meaning.

When analysing the forces and displacements at the crack tip (Table 8), we can observe that the crack tip nodes are subjected to a vertical compressive load (*F_y_* = −0.5 N). The standard VCCT furnishes *G_I_* < 0 because *F_y_* and Δ*v* present opposite signs. However, in this case, the crack tip is subjected to a compressive force; therefore, when the vertical constraint is released, it cannot open the crack in mode I. As a negative *G_I_* value is unrealistic, the *G_I_* value must be corrected and set to *G_I_* = 0. In any case, the *G_I_* value can be neglected since it only represents 0.1% of the *G_II_* value.

#### 3.2.2. Revised I–II VCCT

This subsection describes the solution of the FEM model following the revised I–II VCCT. The results are provided in Table 9.

As can be seen in Table 9, *Fy^a^* and *Fy^b^* are compression forces. In step (a), when the nodes at the crack tip are constrained in both the vertical and horizontal directions, the crack tip nodes are subjected to compression. In step (b), once the vertical constraint is released, *Fy^b^* does not reduce to zero. Instead, a residual compressive force is maintained because of the contact pressure between both arms of the sample. Furthermore, Δ*v^b^* < 0, indicating mesh interpenetration, which it is not physically possible. This may be due to the FEM software’s own contact-detection algorithm, where some mesh interpenetration is necessary to generate the reaction force. Therefore, Δ*v^b^* and *G_I_* must be set equal to zero, in this step.

In the last step (c), *G_I_* < 0 because of the small residual negative force *F_y_^b^* due to the contact between the specimen arms and the small vertical displacement Δ*v^c^*. Again, *G_I_* must be set to zero because the contact force cannot open the crack.

**Figure 11:**
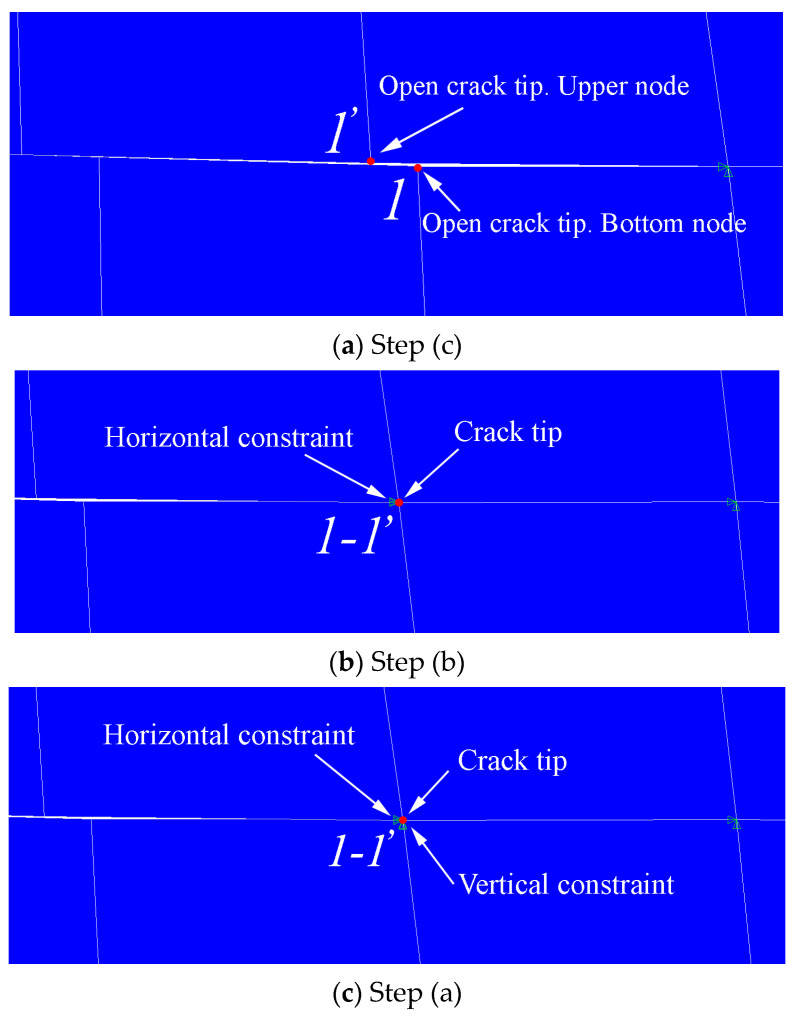
AENF specimen with the lower arm stiffer than the upper arm. Revised I–II VCCT in the closing direction. Deformed shape with a magnification factor = 2. Midpoint applied displacement *δ* = 2. (**a**) Step (c): Crack tip is initially open. Nodes 1 and 1′ are not coupled together (the red dots represent the nodes). (**b**) Step (b): Horizontal closure, Δ*u^b^* = 0. Nodes 1 and 1′ are coupled horizontally but are free to move vertically (blue triangle indicate the horizontal coupling). When this restriction is imposed, the crack tip also closes vertically in this case. (**c**) Step (a): Vertical closure Δ*v^b^* = 0 while maintaining Δ*u^b^* = 0. Nodes 1–1′ are now joined together (blue triangles indicate the horizontal and vertical couplings).

We can also analyse this process in the closing direction, from step (c) to step (a), as shown in Figure 11. The final crack tip is completely open in step (c) (Figure 11c). In the next step, the initial small gap between the nodes is closed horizontally Δ*u^c^* = −3.48 μm (Figure 11b). However, by this horizontal closing action, not only the horizontal gap is closed, but also the vertical gap, and even a contact pressure equivalent to *F_y_^b^* = −0.16 N is developed, together with an unrealistic node interpenetration of Δ*v^b^* = −0.08 μm. In the last step, Δ*v^b^* = 0 is imposed and the compressive nodal force ramps to *F_y_^b^* = −0.54 N (Figure 11a). This last step is not necessary, as the crack tip has already been closed vertically in the previous step. As a result, *G_I_* must be set equal to zero (*G_I_* = 0) in both steps, and only pure mode II is then produced. As mentioned above, in any case, the *G_I_* value can be neglected as it represents only 0.1% of the *G_II_* value and falls within the possible margin of error of the numerical calculation.

Valvo also studied the VCCT in situations where contact and mesh interpenetration occurred [24]. He developed expressions for cases involving interpenetrated crack in compression and open crack in compression and concluded that, in both cases, *G_I_* = 0.

#### 3.2.3. Revised II–I VCCT

The results are presented in Table 10. As we can see in this Table, *G_I_* takes a very small positive value in the last step. This value is negligible compared to the value of *G_II_*, as it represents only 0.1% of the *G_II_* value. Therefore, as expected, pure mode II occurs in this test configuration.

Table 11 shows a summary of all results. As can be seen in this table, as pure mode II is produced, all three procedures provide similar results (negative *G_I_* values have been set to zero).

### 3.3. AENF Specimen with Upper Arm Stiffer than Lower Arm

Next, the AENF specimen is reversed, placing the stiffer laminate on the top. In this case, a mixed mode occurs at the crack tip [9]. The dimensions of the model are as follows:

- *L* = 100 mm;
- *a*_0_ = 40 mm;
- *h*_1_ = 1 mm;
- *h*_2_ = 5 mm.

A displacement of *δ* = 2 mm is applied to the specimen midpoint. The obtained results are discussed below.

#### 3.3.1. Standard Two-Step VCCT

Table 12 shows the obtained results when applying the standard two-step VCCT.

As observed in Table 12, the specimen now presents a mixed mode at the crack tip.

#### 3.3.2. Revised I–II VCCT

The FEM model was solved following the revised I–II VCCT, and the results are presented in Table 13.

We can see in Table 13 that, as in the ADCB specimen, when the vertical coupling of the nodes at the crack tip is released (*F_y_^b^* = 0) (step (a) to (b)), the horizontal force increases from −49.7 to −51.5 N. This allows an energy transfer from mode I to mode II in the next step ((b) to (c)), when the horizontal coupling is released.

#### 3.3.3. Revised II–I VCCT

The results are presented in Table 14.

As we can see in Table 14, when the horizontal coupling of the nodes at the crack tip is released (*F_x_^b^* = 0), an increase in the vertical force occurs (*F_y_^i^* changes from 16.1 to 17.1 N), which allows for a subsequent increase in the *G_I_* value in the next step when the vertical coupling is released.

Table 15 shows a summary of all three procedures.

As in the previous cases, the standard two-step VCCT furnishes results that are between those of the revised I–II and revised II–I VCCTs. Again, in the revised I–II model, part of the mode I ERR is transferred to mode II, while in the revised II–I VCCT, the ERR transfer occurs from mode II to mode I.

As in the ADCB specimen, the ERR transfer between *G_I_* and *G_II_* in the revised procedures (considering the standard procedure as a reference) can be obtained by Equations (14)–(17).

#### 3.3.4. Influence of the Degree of Asymmetry

**Figure 12:**
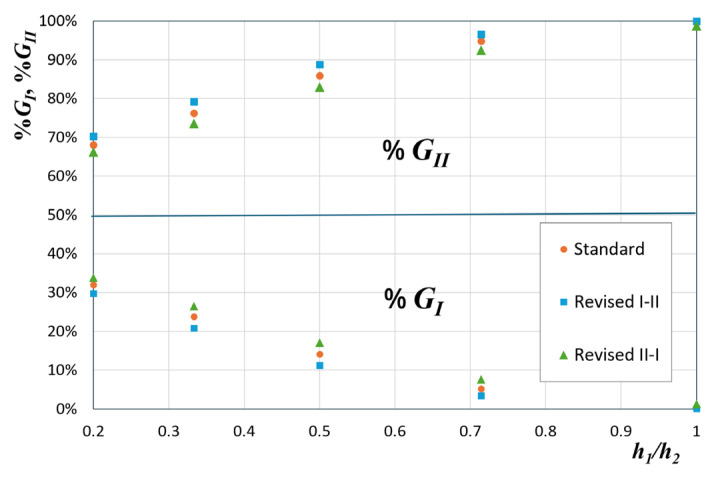
AENF test. Influence of the degree of asymmetry.

Some AENF models were prepared with different degrees of asymmetry. In all models, a displacement of 2 mm was applied to the midpoint of the test specimens. The partition values were calculated using the three VCCTs. Figure 12 shows the obtained results. As can be seen in this figure, for symmetric specimens, the results of the three calculation procedures were coincident. For asymmetric specimens, there was a slight difference between the partition calculations, with the results of the standard procedure falling between the values of the revised procedures.

## 4. Conclusions

In this work, the standard two-step and revised I–II and II–I VCCTs were studied using two asymmetric test configurations (ADCB and AENF). It has been observed in the scientific literature that, when using the standard VCCT on highly asymmetric specimens and under some specific loading conditions, unrealistic negative components of the ERR may be obtained. The revised I–II and II–I VCCTs proposed by Valvo and Wang et al. established alternative ERR partitions with energetically orthogonal components that solve this inconsistency.

It was shown for the studied ADCB and AENF test configurations that the standard two-step VCCT provided mode I and mode II ERR components (*G_I_*, *G_II_*) that were between the equivalent quantities calculated using the revised I–II and II–I models. In the revised I–II model, a certain amount of ERR was transferred to mode II, while in the revised II–I procedure, the energy transfer occurred in the opposite direction, i.e., from mode II to mode I. Therefore, the total ERR produced by the three procedures was the same. This was the expected result, given that the initial and final steps of the models were the same in all three procedures.

When the pure mode occurred, the three procedures provided the same result. When mixed modes occurred, these procedures provided slightly different partition results, with the standard two-step results falling between the revised I–II and II–I results for the ADCB and AENF test configurations studied.

Although the revised VCCTs were expected to always provide positive *G_I_* and *G_II_* values, even these procedures could provide slightly negative *G_I_* values when contact occurred between the specimen arms in the vicinity of the crack tip. In this case, the crack tip was subjected to compression. FEM calculations could also lead to unrealistic slight mesh interpenetration (Δ*v^b^* < 0), probably due to the FEM software’s own contact-detection algorithm, where some mesh interpenetration is necessary to generate the reaction force. As the compression forces cannot open the crack, *G_I_* had to be set to zero in these cases. In any case, the negative values of *G_I_* found in this work were very small, as they represented only 0.1% of the *G_II_* value.

The revised models effectively avoided the interactions between the vertical forces and horizontal displacements and, conversely, those between the horizontal forces and vertical displacements.

Finally, the revised I–II and II–I models not only furnished positive mode I and mode II components of ERR but also decomposed the VCCT into a two-step procedure where successive pure mode I and pure mode II (or vice versa) were produced. As a pure mode was produced in each step, the ERR modes I and II could be easily calculated from the variation in the internal energy in each step, as all the energy variation could be attributed exclusively either to mode I or mode II.

Regarding future work, it is necessary to continue working to improve analytical and numerical formulations to achieve always-positive mode partitions that are consistent and have a solid physical meaning in the description of delamination fracture in composite materials. In this sense, it may be interesting to take into account the rotation of the specimen in the calculation of the mixed-mode decomposition.

## Figures and Tables

**Table 1:** Elastic properties of the laminate used in the FEM models.

*E_x_* (MPa)	*E_y_* (MPa)	*E_z_* (MPa)	*G_xy_* (MPa)	*G_xz_* (MPa)	*G_yz_* (MPa)	*v_xy_*	*v_xy_*	*v_xy_*
144,000	10,600	10,600	5360	5360	3786	0.34	0.34	0.40

**Table 2:** ADCB ERR results. Standard two-step VCCT and variation in internal energy (error shown in brackets).

*F_x_* (N)	*F_y_* (N)	Δ*u* (µm)	Δ*v* (µm)	*G_I_* (N/m)	*G_II_* (N/m)	*G* (N/m)	−Δ*U*/*B*Δ*a* (N/m)
−9.40	11.89	−0.58	2.97	352.8	54.5	407.3	408.5 (0.3%)

**Table 3:** ADCB. Revised I–II VCCT.

**Step (a) to (b)** Pure mode I	***F_x_^a^*** **(N)**	***F_y_^a^*** **(N)**	***F_x_^b^*** **(N)**	***F_y_^b^*** **(N)**	**Δ*u^b^*** **(µm)**	**Δ*v^b^*** **(µm)**	***G_I_*** **(N/m)**	***G_II_*** **(N/m)**
	−9.40	11.89	−10.22	0.00	0.00	2.93	348.3	0.0
**Step (b) to (c)** Pure mode II	***F_x_^b^*** **(N)**	***F_y_^b^*** **(N)**	***F_x_^c^*** **(N)**	***F_y_^c^*** **(N)**	**Δ*u^c^*** **(µm)**	**Δ*v^c^*** **(µm)**	***G_I_*** **(N/m**)	***G_II_*** **(N/m)**
	−10.22	0.00	0.00	0.00	−0.58	2.97	0.0	59.2

**Table 4:** ADCB. Comparison between the standard two-step and revised I–II VCCT.

Procedure	*G_I_* (N/m)	*G_II_* (N/m)	*G* (N/m)
Revised I–II VCCT	348.3	59.2	407.5
Standard VCCT	352.8	54.5	407.3
Difference between revised I–II and standard VCCT	−4.6	4.7	0.2 (0.0%)

**Table 5:** ADCB. Revised II–I VCCT.

**Step (a) to (b)** Pure mode II	***F_x_^a^*** **(N)**	***F_y_^a^*** **(N)**	***F_x_^b^*** **(N)**	***F_y_^b^*** **(N)**	**Δ*u^b^*** **(µm)**	**Δ*v^b^*** **(µm)**	***G_I_*** **(N/m)**	***G_II_*** **(N/m)**
	−9.40	11.89	0.00	12.03	−0.54	0.00	0.0	50.5
**Step (b) to (c)** Pure mode I	***F_x_^b^*** **(N)**	***F_y_^b^*** **(N)**	***F_x_^c^*** **(N)**	***F_y_^c^*** **(N)**	**Δ*u^c^*** **(µm)**	**Δ*v^c^*** **(µm)**	***G_I_*** **(N/m**)	***G_II_*** **(N/m)**
	0.00	12.03	0.00	0.00	−0.58	2.97	357.0	0.0

**Table 6:** ADCB. Comparison between the standard two-step and revised II–I VCCT.

Procedure	*G_I_* (N/m)	*G_II_* (N/m)	*G* (N/m)
Revised II–I	357.0	50.5	407.5
Standard	352.8	54.5	407.3
Difference revised II–I and standard VCCT	4.1	−4.0	0.2 (0.0%)

**Table 7:** ADCB. Summary of the standard two-step, revised I–II, and revised II–I VCCT results. Δ is the percentage difference between the standard two-step and revised VCCT.

Procedure	*G_I_* (N/m)	Δ (%)	*G_II_* (N/m)	Δ (%)	*G* (N/m)
Revised I–II VCCT	348.3	−1.3%	59.2	8.7%	407.5
Standard VCCT	352.8		54.5		407.3
Revised II–I VCCT	357.0	1.2%	50.5	−7.3%	407.5
Average values of revised I–II and II–I	352.6		54.9		407.5

**Table 8:** AENF. Standard two-step VCCT results and variation in internal energy (error shown in brackets).

*F_x_* (N)	*F_y_* (N)	Δ*u* (µm)	Δ*v* (µm)	*G_I_* (N/m)	*G_II_* (N/m)	−Δ*U*/*B*Δ*a* (N/m)
−61.71	−0.54	−3.48	0.27	−1.5	2149.6	2108.6 (−1.8%)

**Table 9:** AENF. Revised I–II VCCT.

**Step (a) to (b)** Pure mode I	***F_x_^a^*** **(N)**	***F_y_^a^*** **(N)**	***F_x_^b^*** **(N)**	***F_y_^b^*** **(N)**	**Δ*u^b^*** **(µm)**	**Δ*v^b^*** **(µm)**	***G_I_*** **(N/m)**	***G_II_*** **(N/m)**
	−61.71	−0.54	−61.65	−0.16	0.00	−0.08	0.3	0.0
**Step (b) to (c)** Pure mode II	***F_x_^b^*** **(N)**	***F_y_^b^*** **(N)**	***F_x_^c^*** **(N)**	***F_y_^c^*** **(N)**	**Δ*u^c^*** **(µm)**	**Δ*v^c^*** **(µm)**	***G_I_*** **(N/m**)	***G_II_*** **(N/m)**
	−61.71	−0.16	0.00	0.00	−3.48	0.27	−0.6	2147.7

**Table 10:** AENF. Revised II–I VCCT.

**Step (a) to (b)** Pure mode II	***F_x_^a^*** **(N)**	***F_y_^a^*** **(N)**	***F_x_^b^*** **(N)**	***F_y_^b^*** **(N)**	**Δ*u^b^*** **(µm)**	**Δ*v^b^*** **(µm)**	***G_I_*** **(N/m)**	***G_II_*** **(N/m)**
	−61.71	−0.54	0.00	1.18	−3.48	0.00	0.0	2145.4
**Step (b) to (c)** Pure mode I	***F_x_^b^*** **(N)**	***F_y_^b^*** **(N)**	***F_x_^c^*** **(N)**	***F_y_^c^*** **(N)**	**Δ*u^c^*** **(µm)**	**Δ*v^c^*** **(µm)**	***G_I_*** **(N/m**)	***G_II_*** **(N/m)**
	0.00	1.18	0.00	0.00	−3.48	0.27	3.3	0

**Table 11:** AENF. Summary of the standard two-step, revised I–II, and revised II–I VCCT results.

Procedure	*G_I_* (N/m)	*G_II_* (N/m)	*G* (N/m)
Revised I–II VCCT	0	2147.7	2147.7
Standard VCCT	0	2149.6	2149.6
Revised II–I VCCT	3.3	2148.7	2148.7

**Table 12:** AENF. ERR results. Standard two-step VCCT and variation in internal energy (error shown in brackets).

*F_x_* (N)	*F_y_* (N)	Δ*u* (µm)	Δ*v* (µm)	*G_I_* (N/m)	*G_II_* (N/m)	*G* (N/m)	−Δ*U*/*B*Δ*a* (N/m)
−49.73	16.14	−2.95	4.27	689.5	1468.7	2158.2	2138.6 (−0.9%)

**Table 13:** AENF. Revised I–II VCCT.

**Step (a) to (b)** Pure mode I	***F_x_^a^*** **(N)**	***F_y_^a^*** **(N)**	***F_x_^b^*** **(N)**	***F_y_^b^*** **(N)**	**Δ*u^b^*** **(µm)**	**Δ*v^b^*** **(µm)**	***G_I_*** **(N/m)**	***G_II_*** **(N/m)**
	−49.73	16.14	−51.48	0.00	0.00	3.99	643.4	0.0
**Step (b) to (c)** Pure mode II	***F_x_^b^*** **(N)**	***F_y_^b^*** **(N)**	***F_x_^c^*** **(N)**	***F_y_^c^*** **(N)**	**Δ*u^c^*** **(µm)**	**Δ*v^c^*** **(µm)**	***G_I_*** **(N/m**)	***G_II_*** **(N/m)**
	−51.48	0.00	0.00	0.00	−2.95	4.27	0.0	1520.4

**Table 14:** AENF. Revised II–I VCCT.

**Step (a) to (b)** Pure mode II	***F_x_^a^*** **(N)**	***F_y_^a^*** **(N)**	***F_x_^b^*** **(N)**	***F_y_^b^*** **(N)**	**Δ*u^b^*** **(µm)**	**Δ*v^b^*** **(µm)**	***G_I_*** **(N/m)**	***G_II_*** **(N/m)**
	−49.73	16.14	0.00	17.11	−2.88	0.00	0.0	1433.2
**Step (b) to (c)** Pure mode I	***F_x_^b^*** **(N)**	***F_y_^b^*** **(N)**	***F_x_^c^*** **(N)**	***F_y_^c^*** **(N)**	**Δ*u^c^*** **(µm)**	**Δ*v^c^*** **(µm)**	***G_I_*** **(N/m**)	***G_II_*** **(N/m)**
	0.00	17.11	0.00	0.00	−2.95	4.27	730.9	0.0

**Table 15:** AENF. Summary of the standard two-step, revised I–II, and revised II–I VCCT results. Δ is the difference in N/m and percentage between the standard two-step and revised VCCTs.

Procedure	*G_I_* (N/m)	Δ (N/m)	Δ (%)	*G_II_* (N/m)	Δ (N/m)	Δ (%)	*G* (N/m)
Revised I–II VCCT	643.4	−46.1	−6.7%	1520.4	51.7	3.5%	2163.8
Standard VCCT	689.5			1468.7			2158.2
Revised II–I VCCT	730.9	41.4	6.0%	1433.2	−35.5	−2.4%	2164.1
Average of I–II and II–I values	687.2			1476.8			2163.8

## Data Availability

The original contributions presented in this study are included in the article. Further inquiries can be directed to the corresponding author.

## Abbreviations

The following abbreviations are used in this manuscript:

FEM	Finite Element Modelling
VCCT	Virtual Crack-Closure Technique
ADCB	Asymmetric Double Cantilever Beam
AENF	Asymmetric End-Notched Flexure
